# Non-contrast-enhanced magnetic resonance imaging can be used to assess renal cortical and medullary volumes—A validation study

**DOI:** 10.1177/20584601211072281

**Published:** 2022-01-21

**Authors:** Jonas Liefke, Katarina Steding-Ehrenborg, Daniel Asgeirsson, David Nordlund, Sascha Kopic, Eva Morsing, Erik Hedström

**Affiliations:** Department of Clinical Sciences Lund, 5193 Lund University, Lund, Sweden

**Keywords:** Renal parenchymal volume, renal cortical volume, renal medullary volume, magnetic resonance imaging, observer variability, validation

## Abstract

**Background:**

Magnetic resonance imaging (MRI) biomarkers can diagnose and prognosticate kidney disease. Renal volume validation studies are however scarce, and measurements are limited by use of contrast agent or advanced post-processing.

**Purpose:**

To validate a widely available non-contrast-enhanced MRI method for quantification of renal cortical and medullary volumes in pigs; investigate observer variability of cortical and medullary volumes in humans; and present reference values for renal cortical and medullary volumes in adolescents.

**Materials and Methods:**

Cortical and medullary volumes were quantified from transaxial in-vivo water-excited MR images in six pigs and 15 healthy adolescents (13–16years). Pig kidneys were excised, and renal cortex and medulla were separately quantified by the water displacement method. Both limits of agreement by the Bland-Altman method and reference ranges are presented as 2.5–97.5 percentiles.

**Results:**

Agreement between MRI and ex-vivo quantification were -7 mL (-10–0 mL) for total parenchyma, -4 mL (-9–3 mL) for cortex, and -2 mL (-7–2 mL) for medulla. Intraobserver variability for pig and human kidneys were <5% for total parenchyma, cortex, and medulla. Interobserver variability for both pig and human kidneys were ≤4% for total parenchyma and cortex, and 6% and 12% for medulla. Reference ranges indexed for body surface area and sex were 54–103 mL/m^2^ (boys) and 56–103 mL/m^2^ (girls) for total parenchyma, 39–62 mL/m^2^ and 36–68 mL/m^2^ for cortex, and 16–45 mL/m^2^ and 17–42 mL/m^2^ for medulla.

**Conclusion:**

The proposed widely available non-contrast-enhanced MRI method can quantify cortical and medullary renal volumes and can be directly implemented clinically.

## Introduction

The global health burden of renal dysfunction is substantial and increasing.^1^ Renal dysfunction also increases risk for severe cardiovascular complications,^2–5^ and is one of the most important risk factors for poor outcome in patients with heart failure.^6^ In clinical practice, renal macroscopic changes are commonly assessed by ultrasound. Albeit widely available, ultrasound has high intra- and interobserver variability and may underestimate renal volumes with up to 48%.^7–9^ A more accurate and precise method for quantifying renal volumes can potentially aid in evaluation of renal dysfunction, in addition to traditional measures of glomerular filtration rate and albuminuria. It may also help predict future renal functional decline and associated morbidity in a variety of diseases.^10–14^

Contrast-enhanced magnetic resonance imaging (MRI) and computed tomography (CT) can be used for accurate quantification of renal parenchymal volumes.^9,14–16^ Due to potential contrast agent toxicity, especially in patients with renal dysfunction, non-contrast-enhanced imaging is preferred.^17,18^ Discrimination between cortex and medulla is challenging in non-contrast-enhanced CT images. Previous non-contrast-enhanced MRI techniques apply either specialized MRI sequences or advanced post-processing techniques,^19,20^ and the methods are thus not widely available. Validated and widely available non-contrast-enhanced MRI methods for volume quantification of renal cortex and medulla are also lacking.

The aims of this study were therefore to validate a widely available non-contrast-enhanced MRI method for quantification of renal cortical and medullary volumes in pigs; investigate observer variability of cortical and medullary volumes in humans; and present reference values for renal cortical and medullary volumes in adolescents.

## Material and Methods

The Regional Ethical Review Board approved this prospective study. All adolescents, and guardians when applicable, gave written informed consent before participating. The study was performed according to animal care guidelines and the Declaration of Helsinki.

### Experimental study

Twelve kidneys were imaged in vivo in six pigs (weight 44–47 kg). The animals were pre-medicated with Ketamine 15 mg/kg (Ketaminol, Intervet, Danderyd, Sweden) and Midazolam 0.5 mg/kg intramuscularly (Dormicum, Roche AB, Stockholm, Sweden) after overnight fasting with free access to water. General anesthesia was induced with Propofol 20 mg/kg (Propofol Sandoz AS, Copenhagen, Denmark) and animals were intubated with a cuffed endotracheal tube. General anesthesia was maintained with Isoflurane (Isoflurane, Baxter Medical AB, Kista, Sweden) using a disposable administration system (Anaconda, Sedana Medical AB, Uppsala, Sweden). Animals were mechanically ventilated using volume-controlled mode regulated towards a pCO_2_ of 5–6 kPa. A 5% glucose infusion, 0.9% NaCl infusion, norepinephrine, and fentanyl were administered as needed.

After MRI, still under general anesthesia, the animals were euthanized with a rapid infusion of potassium chloride. Abdominal incision was performed, and the renal vessels and ureters were immediately clamped to decrease volume changes during excision. Kidneys were excised and marked right/left. Parenchymal tissue, that is, medulla and cortex, were dissected and quantified separately using the reference standard water displacement method (100 mL volumetric flask with 2 mL increments, tolerance ±2.0 mL at 20°C) according to Archimedes’ principle.^21^ All pig kidneys were included for analysis of observer variability.

### Adolescents

Forty-eight kidneys were imaged in 24 healthy adolescents of median age 14 years (range 13–16; 54% females). Healthy adolescents had no history of renal disease, hypertension, or vascular disease. A subset of 12 human kidneys was randomly chosen for analysis of observer variability. In humans, systolic and diastolic blood pressures were measured in the right upper arm using an automatic sphygmomanometer during the MR examination. Estimated glomerular filtration rate (eGFR) was calculated using the CAPA (Caucasian, Asian, Pediatric and Adult) equation based on Cystatin C.^22^ Body surface area (BSA) was calculated using the Mosteller formula.^23^

### Magnetic resonance imaging and image analysis

In-vivo fast low angle shot (FLASH) MR images at 1.5 T (MAGNETOM Aera, Siemens Healthcare, Erlangen, Germany) were acquired as transaxial stacks. Typical parameters were 1.3×1.3×6 mm^3^, TR/TE=152/5.57 ms, FA=80°, bandwidth=270 Hz/px, GRAPPA=2 with 24 reference lines, water excitation and 50 mm saturation bands head/foot with gap 10 mm. Pig kidneys were acquired during free breathing while human kidneys were acquired during breath-hold.

Magnetic resonance images were manually delineated in Segment (version 3.1; Medviso AB, Lund, Sweden) for quantitative measurements.^24^ Three observers with 3 (observer 1), 15 (observer 2), and 20 (observer 3) years of MRI experience, respectively, separately delineated renal cortex and medulla. Delineations were supported by anatomical information in slices above and below the current slice. Cysts, renal pelvis, and other non-parenchymal tissue were excluded. Observer 1 received thorough training in renal segmentation and had experience from two training sets of a total of 20 kidneys with feedback before delineating for the current study.

The more experienced observers provided consensus delineations for interobserver variability whereas observer 1 also assessed intraobserver variability with delineations repeated two months after initial delineations. Initial quantifications by observer 1 were used for comparison with ex-vivo quantification and for interobserver variability. Differences in the medulla to total parenchymal volume ratio between pigs and humans have been reported.^16^ To establish the respective medullary fraction for pig and human in the current study, medullary fraction was calculated as medullary volume/total parenchymal volume.

### Renal volume reference values in adolescence

Reference values for total renal parenchymal volumes, cortical volumes, and medullary volumes in healthy adolescents were calculated as 2.5^th^–97.5^th^ percentiles for boys and girls, both as absolute values and indexed for BSA.

### Statistical analyses

Analyses were performed in GraphPad Prism (version 9.0.1 for Windows, GraphPad Software, San Diego, California USA). Data were visualized in histograms and deemed non-parametric. Subject characteristics are presented as median (range). Agreement between MRI and ex-vivo quantifications and intra- and interobserver variability were evaluated by the Bland-Altman method^25^ and presented as median and 95% limits of agreement (LoA; 2.5^th^–97.5^th^ percentile). Correlation between MRI and ex-vivo quantification was evaluated by Spearman’s rank order correlation. The Wilcoxon signed-rank test and the Mann–Whitney *U* test were performed to test for differences, with *p* values <0.05 considered to show statistically significant differences.

## Results

**Figure 1.**
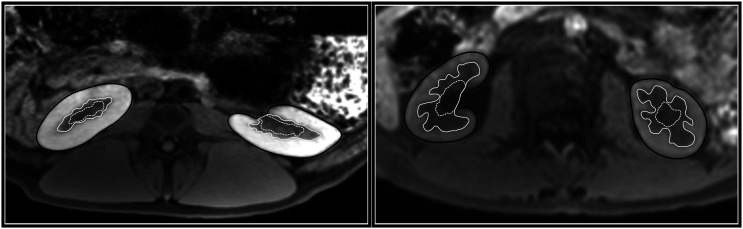
Renal MR images with delineations of renal parenchymal volumes. In-vivo non-contrast-enhanced water-excited fast low angle shot (FLASH) 1.5 T MR images in pig *(left)* and human *(right)* with delineations of cortical and medullary borders. Delineations were supported by anatomical information in slices above and below the herein depicted slices. Cortical outer borders are shown by solid black lines, medullary borders by solid white lines and non-parenchymal regions by dashed white lines.

Figure 1 shows representative image quality in pigs and humans, and delineations of renal parenchymal volumes. Cortex, medulla, and surrounding tissue were well discriminated in all cases and all data sets were used for analysis. Image acquisition including image positioning were approximately 2 minutes and total time for manual segmentation of a pair of kidneys including cortical, medullary, and total volumes was approximately 30 min.

### Experimental study

**Figure 2.**
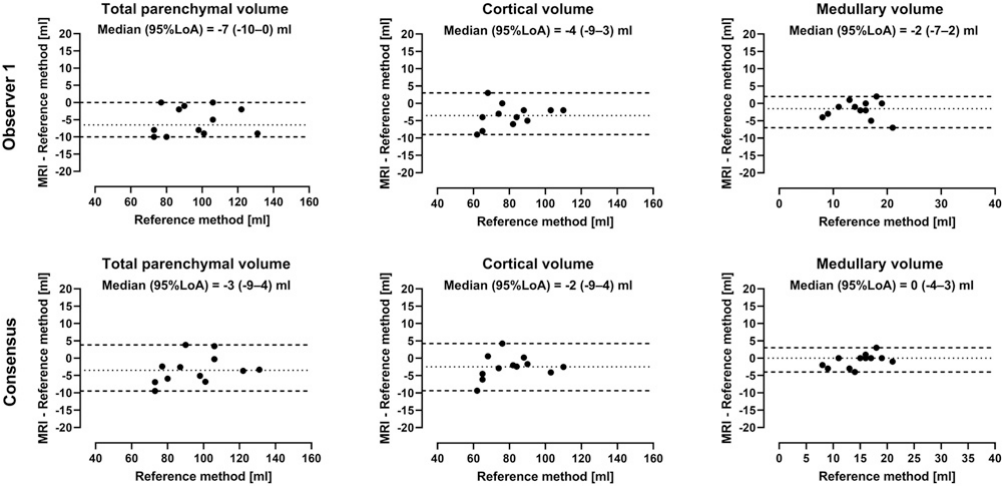
Agreement between renal parenchymal volumes by MR imaging and ex-vivo reference standard. Bland-Altman plots showing agreement for observer 1 *(top)* and consensus observers *(bottom).* Dotted lines indicate median and dashed lines 95% limits of agreement (LoA).

Agreement between MRI and ex-vivo volumes are shown in Figure 2. Agreement between volumes by MRI by observer 1 and ex-vivo quantifications were for total parenchymal volume −7 mL (95% LoA -10–0 mL), for cortical volume −4 mL (95% LoA -9–3 mL), and for medullary volume −2 mL (95% LoA -7–2 mL). Renal volumes by MRI were thus greater than by the water displacement method; total parenchymal volume (6%), cortical volume (4%), and medullary volume (11%; all *p*<0.05). Agreement between MRI volumes by consensus observers and ex-vivo quantification was for total parenchymal volume −3 mL (95% LoA -9–4 mL), for cortical volume −2 mL (95% LoA -9–4 mL), and for medullary volume 0 mL (95% LoA -4–3 mL). Medullary fraction was 15% (range 13–20%).

**Figure 3.**
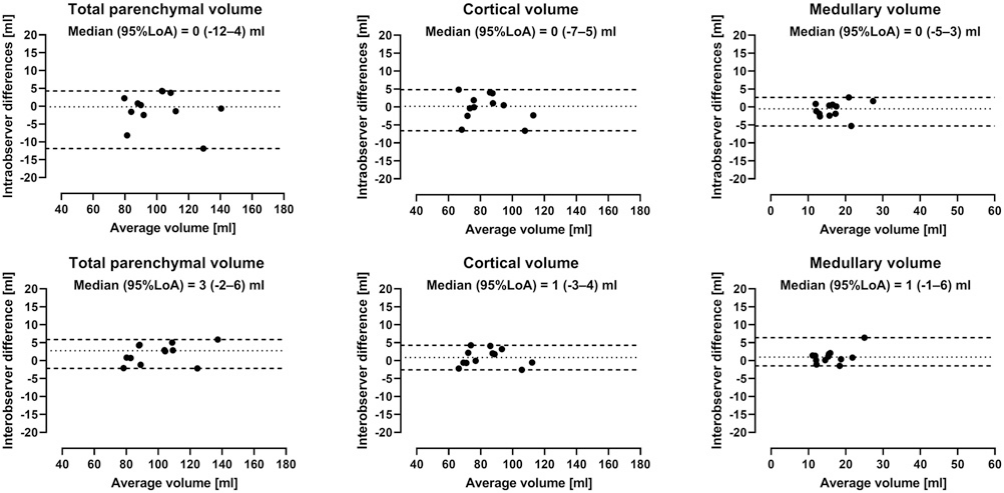
Intra- and interobserver variability of renal parenchymal volumes in pig. Bland-Altman plots showing intra- *(top)* and interobserver *(bottom)* renal parenchymal volumes measured by non-contrast-enhanced MR in pigs. Dotted lines indicate median and dashed lines 95% limits of agreement (LoA).

Intra- and interobserver variability for pig kidneys are shown in Figure 3. Intraobserver variability was for total parenchymal volume 0 mL (95% LoA -12–4 mL), for cortical volume 0 mL (95% LoA -7–5 mL), and for medullary volume 0 mL (95% LoA -5–3 mL). Interobserver variability was for total parenchymal volume 3 mL (95% LoA -2–6 mL), for cortical volume 1 mL (95% LoA -3–4 mL), and for medullary volume 1 mL (95% LoA -1–6 mL).

### Adolescents

Healthy subjects were 14 years (range 13–16), with height 167 cm (range 155–189 cm), weight 58 kg (range 37–89 kg), BMI 20 kg/m^2^ (range 15–25 kg/m^2^), BSA 1.7 m^2^ (1.3–2.2 m^2^), systolic blood pressure 102 mmHg (range 89–130 mmHg) and diastolic blood pressure 50 mmHg (range 44–80 mmHg), and eGFR 100 mL/min/1.73 m^2^ (range 76–122 mL/min/1.73 m^2^).

Table 1 shows renal parenchymal volumes presented as absolute volumes and indexed to BSA for boys and girls. There was no difference in total parenchymal volume between boys and girls, either in absolute values or indexed for BSA (126 mL vs 121 mL, *p*=0.4 and 72 mL/m^2^ vs 76 mL/m^2^, *p*=0.1). Neither was there a difference for cortical volume (87 mL vs 80 mL, *p*=0.1 and 50 mL/m^2^ vs 50 mL/m^2^, *p* = 0.6) nor for medullary volume (36 mL vs 39 mL, *p*=0.4). However, medullary volumes indexed to BSA were different between sexes (21 mL/m^2^ vs 25 mL/m^2^, *p*=0.01). Medullary fraction was in humans 35% (range 27–38%).

**Table 1.** Renal parenchymal volumes presented as absolute volumes (mL) and indexed to body surface area (mL/m^**2**^).

	All (*n* = 24)	Boys (*n* = 11)	Girls (*n* = 13)
	*n* = 48 kidneys	*n* = 22 kidneys	*n* = 26 kidneys
*Renal parenchymal volumes (mL)*			
Total parenchymal volume	122 (80–177)	126 (94–177)	121 (80–154)
Cortical volume	82 (52–133)	87 (66–133)	80 (52–104)
Medullary volume	38 (27–71)	36 (27–71)	39 (27–63)
*Renal parenchymal volumes (mL/m* ^*2*^ *)*			
Total parenchymal volume	75 (54–103)	72 (54–103)	76 (56–103)
Cortical volume	50 (36–68)	50 (39–62)	50 (36–68)
Medullary volume	24 (16–45)	21 (16–45)	25 (17–42)
*Right and left renal parenchymal volumes (mL)*			
Right kidney total parenchymal volume	121 (80–177)	122 (94–177)	120 (80–149)
Left kidney total parenchymal volume	124 (92–167)	127 (99–167)	122 (92–154)
Right kidney cortical volume	78 (52–133)	80 (67–133)	76 (52–104)
Left kidney cortical volume	83 (57–124)	90 (66–124)	82 (57–94)
Right kidney medullary volume	37 (27–58)	36 (27–58)	37 (27–55)
Left kidney medullary volume	40 (30–71)	38 (30–71)	40 (33–63)

Volumes are presented as median (range).

**Figure 4.**
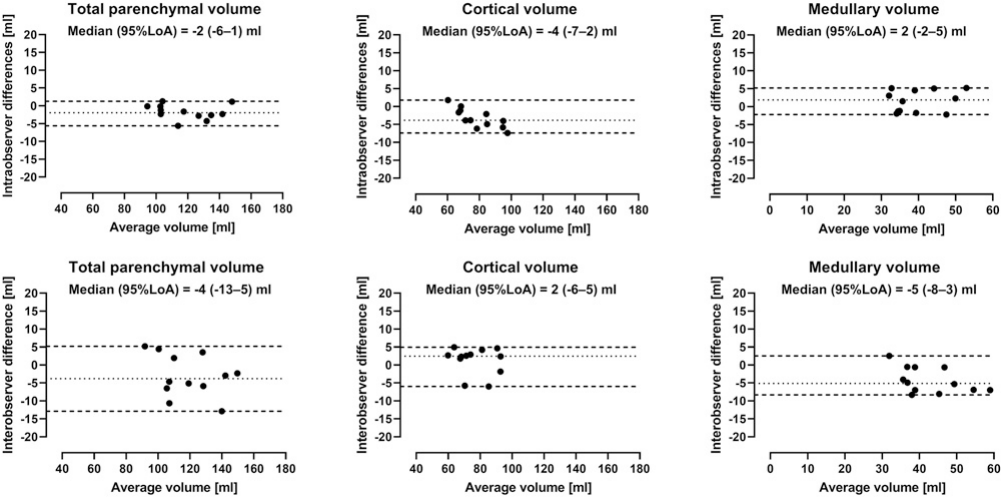
Intra- and interobserver variability of renal parenchymal volumes in human. Bland-Altman plots showing intra- *(top)* and interobserver *(bottom)* renal parenchymal volumes measured by non-contrast-enhanced MR in humans. Dotted lines indicate median and dashed lines 95% limits of agreement (LoA).

Intra- and interobserver variability for human kidneys are presented in Figure 4. Intraobserver variability was for total parenchymal volume −2 mL (95% LoA -6–1 mL), for cortical volume −4 mL (95% LoA -7–2 mL), and for medullary volume 2 mL (95% LoA -2–5 mL). Interobserver variability was for total parenchymal volume −4 mL (95% LoA -13–5 mL), for cortical volume 2 mL (95% LoA -6–5 mL), and for medullary volume −5 mL (95% LoA -8–3 mL).

### Renal volume reference values in adolescents

Table 2 shows reference ranges for renal parenchymal volumes in boys and girls respectively, presented as absolute volumes and indexed to BSA.

**Table 2.** Reference ranges for renal parenchymal volumes presented as absolute volumes (mL) and indexed to body surface area (mL/m^**2**^).

	Boys (*n* = 11)	Girls (*n* = 13)
	*n* = 22 kidneys	*n* = 26 kidneys
*Renal parenchymal volumes (mL)*		
Total parenchymal volume	94–177	80–154
Cortical volume	66–133	52–104
Medullary volume	27–71	27–63
*Renal parenchymal volumes (mL/m* ^*2*^ *)*		
Total parenchymal volume	54–103	56–103
Cortical volume	39–62	36–68
Medullary volume	16–45	17–42

Values are presented as 2.5–97.5 percentiles.

## Discussion

The proposed widely available non-contrast-enhanced MRI method is accurate and precise in quantifying renal cortical and medullary volumes in pigs. Intra- and interobserver variability for quantification of renal parenchymal volumes in pigs and humans were low in the current study. This method can thus reliably assess renal cortical and medullary volumes, increasing applicability of renal imaging for clinical purposes. Further, it enables quantification of renal parenchymal volumes in studies where gadolinium contrast agents are preferred to be avoided, for example, in cardio-renal, transplantation, and pediatric studies.

### Experimental study

The current study is the first to provide validation of medullary volume quantification by non-contrast-enhanced MRI. Further, agreement between MRI and the reference method was in the current study 3–6% for total and cortical renal parenchyma. This is lower or similar compared with previous studies in which either contrast-enhanced MRI or advanced MRI sequences and post-processing were applied, with Coulam et al.^16^ and Cheong et al.^9^ showing ranges of 5–8% and 4–5%, respectively, for total parenchymal volume (bias not reported).

Although showing a low bias, MRI generally overestimated renal parenchymal volumes in the current study. Coulam et al.^16^ also reported overestimation of renal parenchymal volumes by MRI as compared to the water displacement method using correlation (r=0.95). For comparison, correlation between volumes by MRI as compared to the water displacement method in the current study was for total parenchymal volume (r=0.95), for cortical volume (r=0.94) and for medullary volume (r=0.89). An overestimation of renal parenchymal volumes by MRI might be due to a drop in renal parenchymal perfusion pressure after euthanasia. We limited the potential loss of liquids by clamping renal vessels and ureters before excision, but this may be too late to compensate for immediate changes as abdominal surgery was initiated after euthanasia. Fluid alterations after euthanasia could alter tissue volumes and may potentially be avoided by ex-vivo MRI before dissection as a second reference versus the water displacement method, this was however not allowed in the current experimental setup.

### Adolescents

Reference values for renal cortical and medullary volumes by MRI were lacking, why reference ranges are presented in the limited age range included in this study. The current study included healthy adolescents aged 13–16 years for proof of concept for using the proposed MRI method in humans. A complete age range was thus beyond the scope of this study. The wide range of renal volumes presented in the current population, despite the limited age range, indicates that renal volume may be a blunt measure to differentiate between pathology and the normal kidney. This is coherent with the wide reference ranges for renal length measurements by ultrasound.^26^

### Clinical implications

The proposed MRI method requires no post-processing and is accurate and precise in quantifying both cortical and medullary renal volumes. Total analysis time for one patient was approximately 30 min, supporting the use of manual segmentation as it is not necessarily more time consuming than previously proposed automatic methods.^27–30^ Further, the current study also shows that quantification of renal parenchymal volume by MRI can be reliably performed also by less experienced observers, increasing applicability. The current study showed <5% intraobserver variability and between 0–12% interobserver variability for pig and human renal parenchymal volumes. For comparison, Karstoft et al.,^20^ reported ≤5% variability in pig and Will et al.,^27^ and Di Leo et al.,^31^ reported 5–13% in humans. The pig model in the current study showed lower discernibility between cortex and medulla than in healthy volunteers. Therefore, it is likely possible to accurately assess renal parenchymal volumes also in patients where corticomedullary differentiation is lower than in healthy controls. A decrease in discernibility between cortex and medulla is a sensitive but non-specific biomarker of renal dysfunction,^32^ likely related to increased water content with increased MRI T1 relaxation time in renal cortex.^33^ Imaging to study this biomarker has previously been done by administering contrast agents or by acquiring images with advanced post-processing techniques,^16,19^ limiting availability and applicability of methods in patients with renal dysfunction. Although the method proposed in the current study is promising, it may not be directly transferable to renal parenchymal quantification in all patients with renal disease. However, reliable non-contrast-enhanced MRI makes assessment of renal parenchymal volumes in transplant, pediatric, and renal dysfunction patients possible without increasing patient risks otherwise related to ionizing radiation and contrast agents. More importantly, the method may particularly add value in serial assessment of renal parenchymal volume changes, potentially directly impacting clinical management of patients, as this is a field now limited by the use of methods with lower accuracy and higher observer variability.^8,9^

Patients with renal dysfunction are at risk of adverse cardiovascular complications and vice versa.^34,35^ Whereas detailed cardiac morphology and function are commonly assessed by MRI,^36^ renal parenchymal volumes are not. With the proposed non-contrast-enhanced MRI method, both renal cortical and medullary volumes can easily be assessed in conjunction with cardiac MRI extending the examination time with only a few minutes, potentially gaining valuable information for the care of patients with concomitant cardiac and renal diseases. As renal structural abnormalities is one criteria for diagnosing chronic kidney disease^37^ and as changes in renal parenchymal volumes have been proposed as an additional biomarker in the evaluation and prognosis of renal disease^13,14,37^ it is of importance to obtain volumes more accurate and precise than what ultrasound can provide, to properly evaluate this biomarker. Diagnosis and treatment may be further guided using quantitative MRI methods such as mapping and renal blood flow measurements or tissue perfusion to assess structure and function.

In conclusion, this study validated a widely available non-contrast-enhanced MRI method, showing accuracy and precision in quantifying renal cortical and medullary volumes in pigs, with low intra- and interobserver variability in both pigs and humans. The method increases availability of renal volume quantification and can be directly implemented both clinically and in research where contrast agents is preferred to be avoided, such as in patients after transplantation or with renal dysfunction, and in pediatric studies. Presenting reference ranges, although limited, of renal parenchymal volumes for healthy adolescents by MRI is a first step to further enable clinical implementation.
